# Visualization of Genomic Changes by Segmented Smoothing Using an *L*_0_ Penalty

**DOI:** 10.1371/journal.pone.0038230

**Published:** 2012-06-05

**Authors:** Ralph C. A. Rippe, Jacqueline J. Meulman, Paul H. C. Eilers

**Affiliations:** 1 Department of Clinical Epidemiology, Leiden University Medical Center, Leiden, The Netherlands; 2 Institute of Mathematics, Leiden University, Leiden, The Netherlands; 3 Department of Biostatistics, Erasmus Medical Center, Rotterdam, The Netherlands; Université de Nantes, France

## Abstract

Copy number variations (CNV) and allelic imbalance in tumor tissue can show strong segmentation. Their graphical presentation can be enhanced by appropriate smoothing. Existing signal and scatterplot smoothers do not respect segmentation well. We present novel algorithms that use a penalty on the 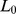 norm of differences of neighboring values. Visualization is our main goal, but we compare classification performance to that of VEGA.

## Introduction

Copy number variations (CNV) and allelic imbalance are common in tumor tissue, reflecting local deviations from diploidy and heterozygosity. When they occur, they typically form segments of widely varying length. As a first step in their analysis, many researchers prefer to have a graphical presentation of genomic changes, as a kind of map along positions on chromosomes. Modern high-density SNP arrays make this possible for hundreds of thousands of positions on the (human) genome.

An array delivers two fluorescence signals for each SNP, one, say *a*, proportional to the dose of one allele, indicated by A, the other, say *b*, proportional to the dose of the other allele, indicated by B. This is only true in principle, because noise and differences between fluorophores of different color can distort the picture to a certain amount. If we ignore these facts for the moment, and consider normal DNA, then the sum of the doses, the copy number, is 2, for any of the genotypes AA, AB or BB. Hence the sum 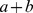 should be almost constant. Similarly the ratio 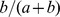 is either 0, 1 or 2; it is called the B allele frequency (BAF). Because in tumor DNA many types of changes can occur, leading to any number of A or B alleles from zero to many, a variety of deviations in CNV and BAF can be found.

**Figure 1:**
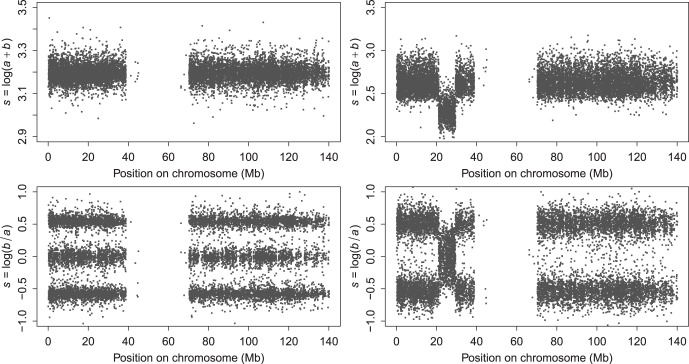
Illustrations of copy numbers and allelic ratio, expressed as logarithms, for healthy and tumor tissue. Left panels: healthy tissue. Right panels: tumor tissue. Top row: copy numbers. Bottom row: allelic imbalance.

We prefer to work with somewhat different combinations of the fluorescence signal. One is the log (to base 10) of their sum, 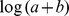, which we abbreviate as LAS (log allelic sum). The reason for working with the logarithm is that usually a quite large range of values of 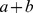 is observed. The other combination is the logarithm of the allelic ratio, 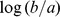, which we will abbreviate as LAR (log allelic ratio). Compared to BAF, LAR strongly expands the scale near 0 and 1, which is crucial when fitting (mixtures of) normal distributions, as we will do in one stage of our data analysis. Figure 1 shows examples of maps of the proposed quantities along chromosome 9 of a normal and a tumor sample.

Copy number analysis has received attention from many investigators; a short overview will follow later in this Introduction. In most cases the aim is to determine, with a solid statistical basis, segment boundaries and copy numbers and allelic doses within the segments. A variety of free and commercial products is available. Yet we believe that there is room for enhanced visualization tools, that allow us to inspect data in some depth before embarking on more formal models. Visualization tools for CNV are widely known, while such tools for allelic imbalance are rare. Therefore, we feel that it is most effective to introduce our new idea in the well-explored field of CNV (LAS) and assess its behavior in depth. Once we have obtained an understanding of its performance, we extend its application to a new setting (LAR), for which there are no “gold standard” comparisons available.

In this paper we present a new approach to copy number smoothing, extending the work of [1]. The main modification is to use a roughness penalty on the number of jumps, instead of on the sum of absolute values of jumps (the 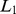 norm). We implement it with an 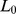 norm, the sum of absolute values of differences raised to the power zero. The result is much sharper segmentation.

Copy number smoothing is relatively simple, because, as the top panels of Figure 1 show, we can interpret the data as one (segmented) trend plus noise. For the allelic ratio the situation is more complicated, because, as the bottom panels show, we can have one, two or three noisy parallel bands. Our solution is to adapt the scatterplot smoother of [2]. In its standard form it computes a histogram on a large two-dimensional grid and applies a smoother on both axes, thus smearing out the counts in both directions. The smoother is based on a penalty on the sum of squares (the 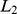 norm) of differences. We apply the same idea, but replace the penalty in the direction along the chromosome with one using the 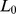 norm. After segmentation with the modified scatterplot smoother, we present the distribution of LAR, separately for each segment, using histograms and Gaussian mixtures.

The literature on segmentation of copy number variations is large. It is a fascinating subject for statistical analysis and it has led to a variety of modeling strategies. We present a short overview of recent work, without claiming completeness.

The hidden Markov model (HMM) is a natural candidate. [3] propose a model with many hidden states, covering copy numbers from zero to seven. They claim improvements compared to older candidates like PennCNV [4] and QuantiSNP [5].

Other models have explicit parameters for the positions of jumps and the levels of the segments between them. VEGA [6] uses dynamic programming, while [7] fit a piecewise linear model by maximum likelihood.

Non-parametric smoothing goes in the opposite direction, by modifying smoothing algorithms in such a way that they favor a piece-wise constant fit. MSMAD [8] is an improvement on the work of [1]. The fused LASSO works in a similar way [9].

Systematic comparisons of a number of models for CNV are available. We mention [10]–[14]. Large-scale assessments over platforms, lab sites and algorithms were made in [15]. The rest of the paper is organized as follows: in Section 2 we present the algorithms, using real data to illustrate them. In Section 3 we compare our segmentation, obtained after automatic selection of the smoothing parameter, with the segmentation from VEGA. In Section 3 we also present applications to clinical samples, including a comparison with segment calls from external software, CNAG [16].

As an acronym for our smoother we use ZEN, derived from Zero Exponent Norm, because the 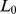 norm in the penalty is crucial to its success.

## Materials and Methods

In this section we first discuss LAS smoothing with penalized least squares, based on several types of norms in the difference penalty. We present a procedure to automatically find a good value for the penalty parameter, using cross-validation. Then we extend the discussion to segmented scatterplot smoothing of LAR. In contrast to smoothing methods that use the sum of squares of absolute values in the norm of the penalty, the objective function of the ZEN smoother is not convex. There is no guarantee that a (unique) global minimum will be reached. Yet in practice we see excellent performance. To increase the confidence of potential users of our methods, we present a short study of convergence behavior.

### Segmented CNV Smoothing

Let the data be *m* data pairs (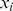, 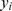), where 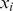 gives the position of SNP *i* (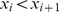 for all *i*) and 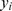 is the copy number signal LAS, 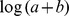, for which we are going to compute a smooth series *z*.

**Figure 2:**
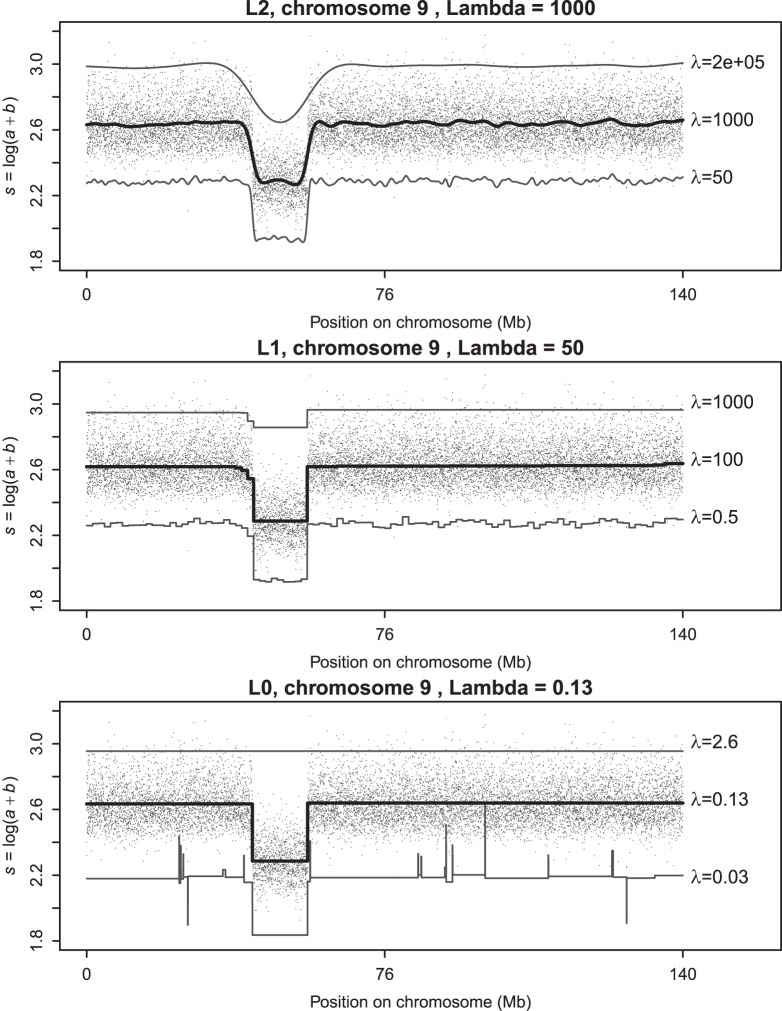
Illustration of smoothing with different norms (2,1,0) in the roughness penalty. Top panel: 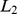 norm, the Whittaker smoother. Middle panel: 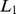 norm. Bottom panel: 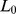 norm. Thinner lines drawn with positive and negative offsets illustrate the effect non-optimal 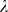. Top line: 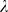 too large. Bottom line: 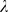 too small.

Our starting point is a variant of the Whittaker smoother [17]. The objective function is
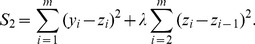
(1)The first term measures fidelity of *z* to *y*, while the second term is a penalty on roughness of *z*. The balance between the two is set by the parameter 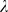; the larger 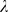 is chosen, the smoother *z* will be. This smoother rounds off edges as is illustrated in the top panel in Figure 2. This is fine in many applications, but not here.

Quantile smoothing replaces the sum of squares (the 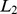 norm) by sums of absolute values (the 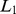 norm). The objective function is
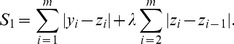
(2)Notice that now fidelity to the data is measured by the sum of the absolute values of 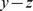 (median smoothing), not by their squares. This modification is necessary because a linear programming algorithm is used to compute 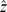. This increases robustness, but decreases sensitivity to the data, compared to the 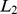 norm. Robustness is hardly an issue in CNV studies.

As can be seen from the middle panel of Figure 2, this modification goes in the right direction. Segments become more clearly visible, but a number of undesirable small jumps occur. We propose the following modification:
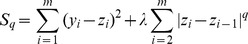
(3)where *q* is a number between 0 and 1. Actually we will concentrate on 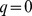, the 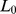 norm. Essentially this is a penalty on the number of non-zero differences between neighboring elements of *z*. Any positive number raised to the power 0 gives 1, while by convention 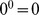. So only non-zero differences add to the penalty, and all by the same amount, independent of their size. Our numerical algorithm approximates this behavior. The lower panel of Figure 2 shows results obtained with the proposed smoother.

**Figure 3:**
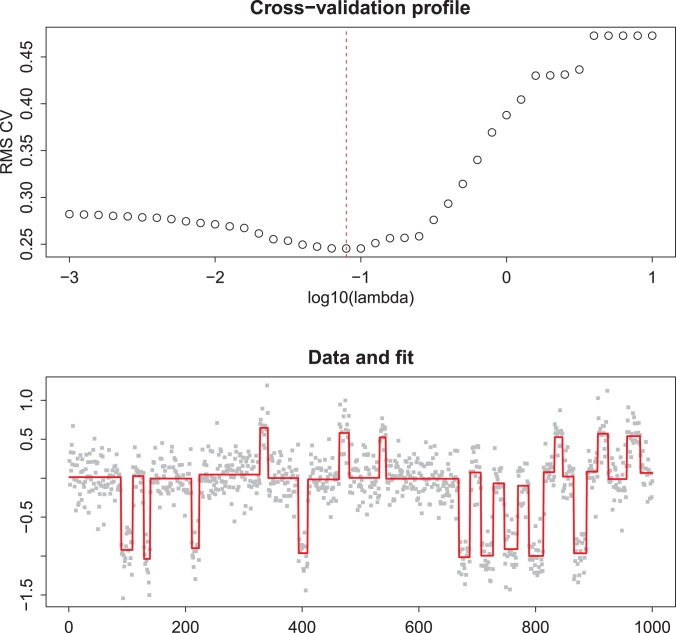
Odd-even cross-validation for finding an optimal 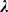. The selected 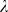 is indicated in the top panel by the vertical broken line. The bottom panel shows data using (double) the selected 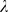 against the raw data. The doubling is needed to compensate for leaving out half of the data.

### Computational Details

It is easy to find the solution for the Whittaker smoother, using matrix-vector operations. If *D* is a matrix that forms first differences, so that if 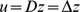, 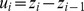, the objective function can be written as 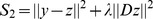, with an explicit solution that follows from the linear system 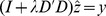. The system is very sparse, which can be exploited in Matlab or R (we use the package spam), leading to computation times that increase linearly with the length of the data series.

We propose a simple, but effective, algorithm to minimize 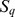, using iterated weights in an adapted Whittaker smoother, borrowing from [18]. It is clear that 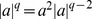, for any number *a*. If we do not know *a* itself, but an approximation 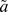, then 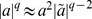. Using this relation, we approximate 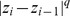 by 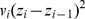, with 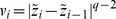. If 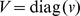, the system to be solved becomes 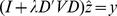. This gives a new approximation to the solution from which new weights are computed. These steps are iterated until convergence.

The function we try to optimize is non-convex, but with decent starting values optimization is effective. However, to improve numerical stability and reduce the number of iterations, we modify the weights somewhat: 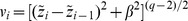, where 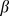 is a small number, of the order of 1/10000th of the expected size of the jumps. If 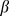 is set not small enough, rounding will occur near the jumps.

**Figure 4:**
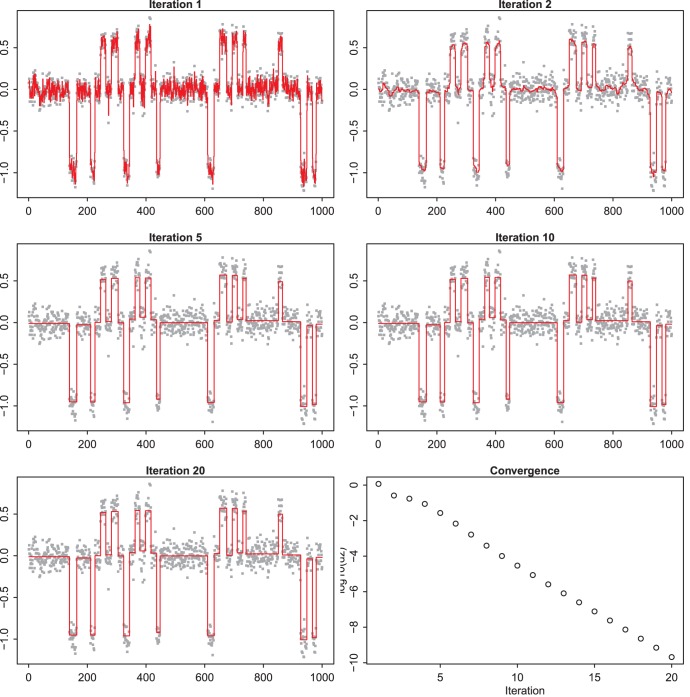
Illustration of convergence behavior in zero-norm smoothing with little noise. The data are simulated (VEGA package) and contain relatively little noise. All panels, except the lower-right one, show intermediate solutions, at the iteration numbers as indicated in the titles of the panels. The lower right panel shows the largest absolute change in the solution at each iteration. The smoothing parameter is set to 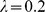.

**Figure 5:**
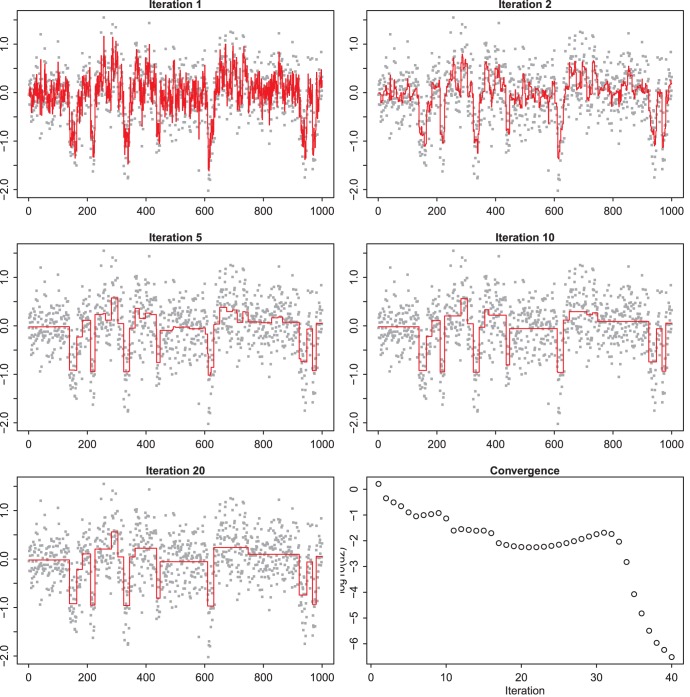
Illustration of convergence behavior in zero-norm smoothing with moderate noise. Illustration of convergence behavior. The data are simulated (VEGA package) and contain relatively much noise. All panels, except the lower-right one show intermediate solutions, at the iteration numbers as indicated in the titles of the panels. The lower right panel shows the largest absolute change in the solution at each iteration. The smoothing parameter is set to 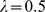.

### Cross-validation for a Good 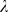


A useful property of the smoother is that it automatically interpolates values for missing observations if we introduce proper weights. The objective function is modified to
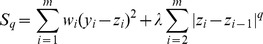
(4)For a missing, or left-out, observation we set 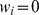; all other weights are set to 1. Smoothly interpolated values for *z* will be computed automatically. The system to be solved in each iteration becomes



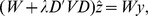
with 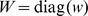.

We exploit this property in cross-validation (CV) to find the optimal smoothing parameter 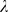. We leave out the even observations, by setting their weights to zero. We then compute
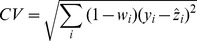
for a series of values of 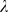 (a linear sequence for 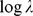) and search for the minimum of CV. This simple cross-validation scheme works well in practice.

Notice that the value of 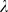 that minimizes CV should be doubled when smoothing the complete data. The value of 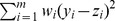 is close to half that of 
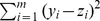
, while the penalty contains all elements of *z* and so will have approximately the same value, whatever the weights.

Applying odd/even cross-validation is effective, as is illustrated in Figure 3. For the cross-validated fit values we observe a clear minimum (top panel), while the smoothed result (bottom panel) looks adequate too, when judged visually.

We don’t want to overstate the importance of cross-validation and optimal smoothing in the present application. Our primary goal is visualization and we expect that the user will play with 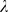 when exploring data. The “optimal” value of 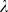 should only be considered an advice. Because the necessary computations take little time on a modern PC, interactive use is possible with attractive speed.

In Section 3 we compare the classification performance of our smoother with that of VEGA, using cross-validation to select 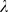.

**Figure 6:**
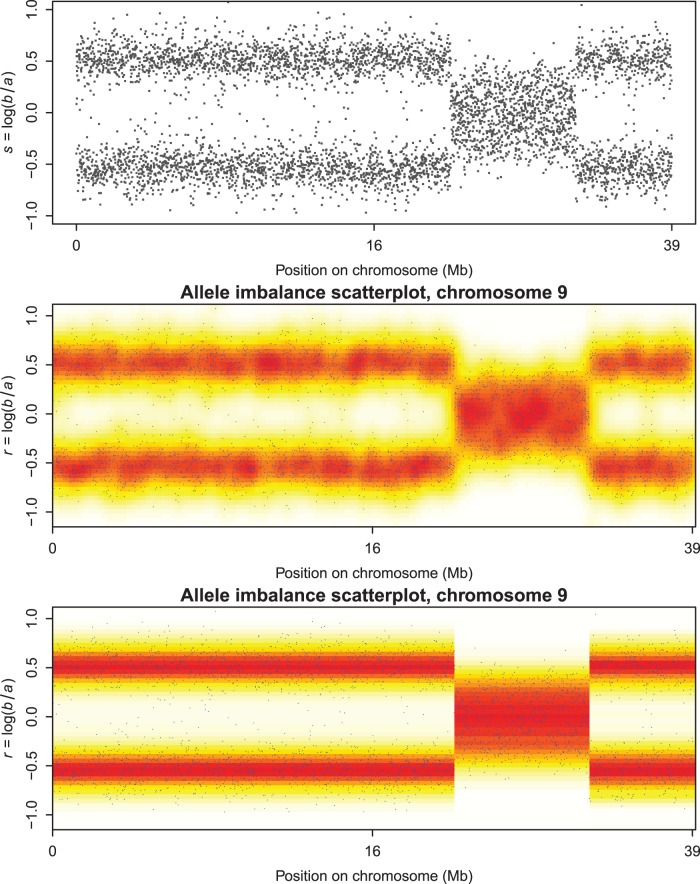
Comparing normal and segmented scatterplot smoothing. Top panel shows the raw observations. Middle panel shows straightforward smoothing: no segmentation. Bottom panel shows segmented smoothing: clear segments.

**Table 1:** Comparing ZEN (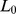) and VEGA on (P)recision, (R)ecall and (F)-value, using simulated data.

	ZEN			VEGA		
*σ*	P	R	F	P	R	F
0.0	1.000	1.000	1.000	1.000	1.000	1.000
0.1	**1.000**	**1.000**	**1.000**	**1.000**	**1.000**	**1.000**
0.2	0.999	**1.000**	0.999	**1.000**	**1.000**	**1.000**
0.3	0.976	0.992	0.984	**0.989**	**0.993**	**0.991**
0.4	0.808	0.938	0.864	**0.911**	**0.953**	**0.931**
0.5	0.797	0.912	0.848	**0.867**	**0.916**	**0.888**
0.6	0.635	**0.821**	**0.709**	**0.675**	0.770	0.706
0.7	0.619	**0.797**	0.687	**0.669**	0.794	**0.721**
0.8	0.601	**0.818**	**0.687**	**0.630**	0.785	0.685
0.9	**0.530**	0.614	0.536	0.469	**0.741**	**0.565**
1.0	**0.485**	0.593	0.514	0.465	**0.752**	**0.559**

### Convergence Behavior

The objective function of the smoother is non-convex, because of the 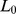 norm in the penalty. Hence there is no guarantee that local minima do not exist, nor that we will always reach a global minimum. Yet in our experience the results make a lot of sense when inspected visually. So even if a solution might not be optimal – and we have no practical means to decide on that – it can be very useful. In this section we present some details on convergence behavior, following the iterations of smoothing with the adaptive weights in the penalty.

Figure 4 presents results for a data set with relatively little noise. They were obtained from the VEGA website [6]. We smooth with 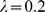 and show the current estimate of the solution *z* at five iteration steps. In the first iteration, all weights, *v*, in the penalty are equal to 1. So effectively we have a light Whittaker smoother. After the first iteration the adaptive weights take effect. As can be seen, after five iterations the final result has almost been reached. The (logarithms) of the change in the solution from one iteration to the next are shown in the lower right panel. The changes are computed as the maximum of the absolute values of the differences.

In this example sufficient convergence was reached quickly, certainly for visualization purposes. In our experience 20 to 40 iterations is typical. Figure 5 shows a noisier data set (also from VEGA), where 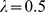. Convergence is slower there.

**Figure 7:**
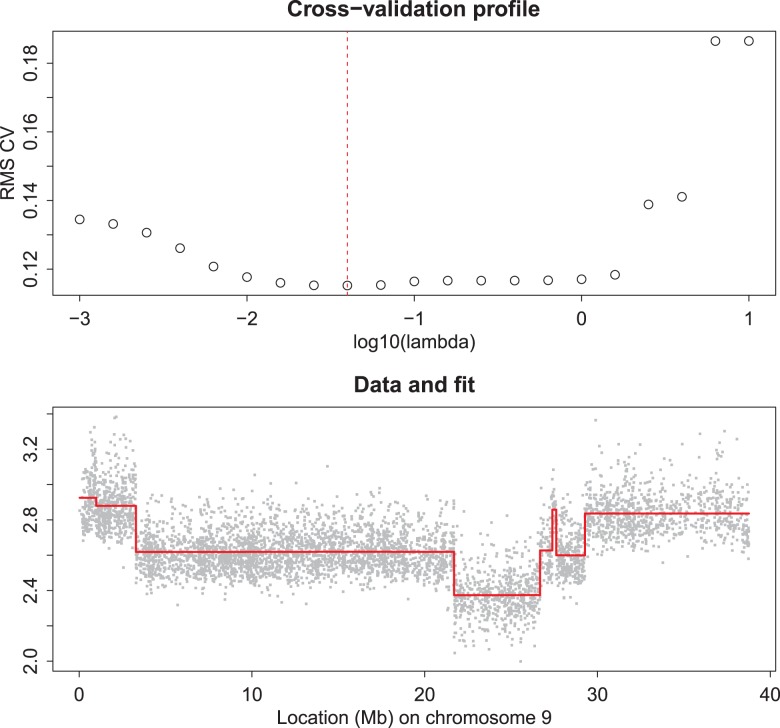
ZEN smoothing of CNV in tumor data (sample GBM139.CEL). Top panel: cross-validation profile and location of minimum (at broken vertical line). Bottom panel: data and fit, using 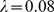 (double the value indicated by cross-validation, to correct for leaving out half of the data).

**Figure 8:**
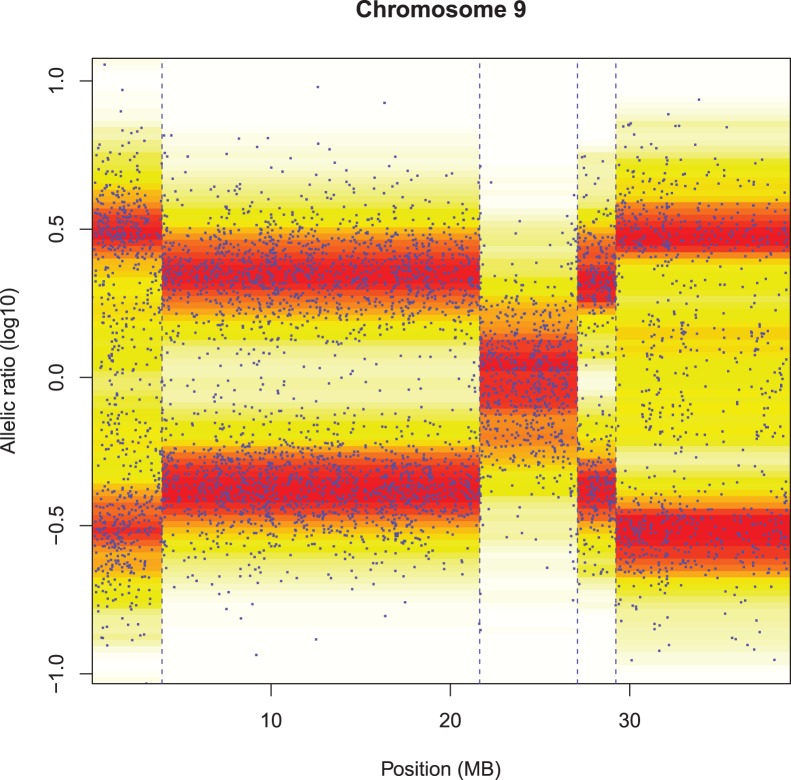
ZEN smoothing of log allelic ratio (sample GBM 139.CEL). The vertical broken lines indicate the segment boundaries, as computed from the adaptive weights in the penalty. The smoothing parameters (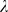) are 0.01 for position and 0.5 for log allelic ratio.

### Segmented Scatterplot Smoothing

A fast smoother for scatterplots was introduced in [2]. The principle is to first compute a two-dimensional histogram on a large grid (say 100 by 100 bins) and to smooth first the columns and then the rows with a Whittaker smoother, having a slightly changed roughness penalty. In order to ensure positive values in the histogram, a combination of a first and second-order penalty is used. If *y* represents one column of the histogram, that will be smoothed to get *z*, the objective function is:
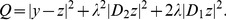
(5)Notice the combination of first (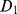) and second order (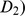 difference penalties. A (banded) linear system of equations results:



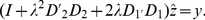
(6)The lower panel of Figure 6 shows results obtained with this smoother, when applied to a scatterplot of (log) allelic ratio against chromosomal position. The raw observations are shown in the top panel. This would be a useful display if it showed sharp segment edges like those we obtained for copy numbers, while maintaining smoothness in the other direction.

**Figure 9:**
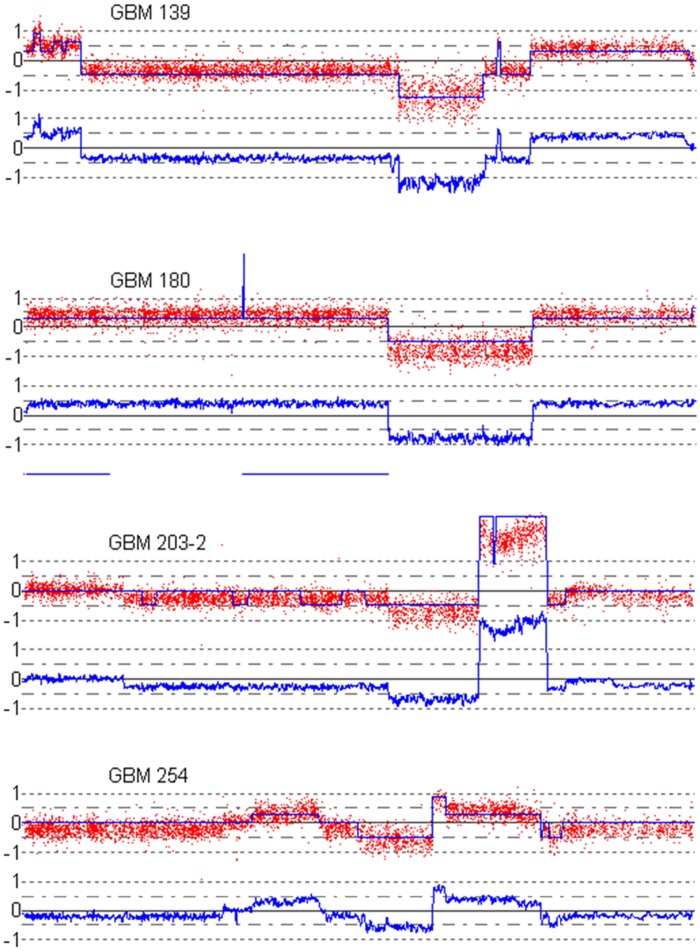
Examples of smoothed CNV and allelic imbance in clinical samples, using ZEN. First and third row show CNV profiles, second and fourth rows show the matching segmented allelic imbalance plots.

**Figure 10:**
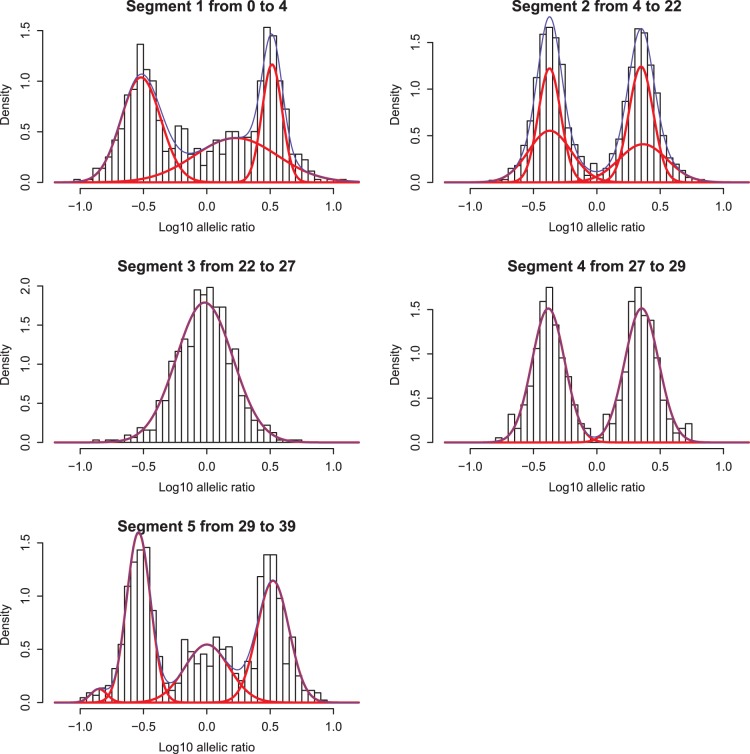
Examples of smoothed CNV in clinical samples, using CNAG software. Panels show CNV profiles for the samples mentioned in the panel titles. The smoothed signals show unexepected jumps (GBM180) and unclear level overestimations (GBM203-2).

**Figure 11:**
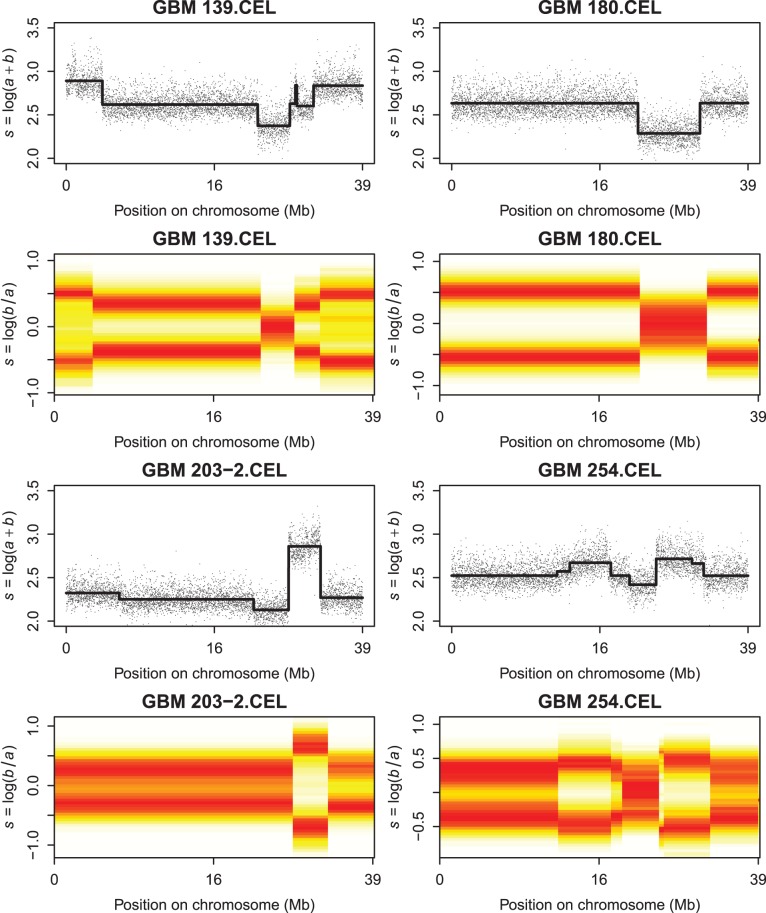
Histograms and estimated normal mixtures for the log allelic ratio. Estimations are separate for each of the five segments that were derived from the scatterplot smoother in Figure 8.

For the segmented scatterplot smoother, we keep the original penalty for the allelic ratio, but for the position we use a penalty based on the 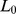 norm of first differences. It will not work to just use that penalty for each row of the histogram: we get segments, but they will generally be in different places for different rows. To avoid it we use the same weight matrix *V* in the penalty 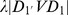, but now compute it as the summary of all rows:
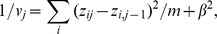
with *m* the number of rows and 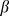 again a small number to increase stability and speed of convergence. Figure 6 (bottom panel) shows a result obtained in this way. Now we get sharp segment boundaries.

A typical vector *v* consists mostly of large numbers and a few small ones. The latter indicate the segment boundaries and these values have been used to enhance the figure with vertical broken lines at the boundaries.

Once the segment boundaries have been found, it makes sense to plot histograms of the (log) allelic ratio for each segment separately. In addition we fit gaussian mixtures using the package mclust [19]. The centers of the mixture components and the difference between them can be used to summarize results and to help the user in interpreting the observed genomic changes. We do not discuss that here, because we feel that that would stray us to far away from our primary goal, visualization.

Like the scatterplot smoother of [2], we see the segmented scatterplot smoother only as a visual aid. We did not try to develop an algorithm for automatic choice of the amount of smoothing, nor did we try to simulate realistic allelic imbalance scenarios to evaluate performance.

## Results

### Simulations

A method for visual segmentation is less useful when it remains unclear whether a correct segmentation is found. In this section we compare performance of our smoother with that of VEGA on CNV segment detection.

We use again the simulated data that are provided by [6]. It contains simulated CNV data for 22 chromosomes, for each of which there are 1000 data points generated. For each chromosome random mutations were generated with a segment length varying between 11 and 25 points. Gain or loss properties for each segment were also randomly selected. Additionally, these data are provided with 10 levels of noise (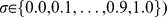, where 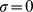 indicates perfect data. We will use these as a reference for segment recovery.

Comparisons between the VEGA method and the proposed 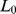 norm smoother are made in terms of *precision*, *recall* and associated *F*-scores. All of these require True Positive Rate (TPR), False Positive Rate (FPR), True Negative Rate (TNR) and the False Negative Rate (FNR). Hits compared to the noise -free data are assessed per individual data point. We define a deviation as at least 1% of the largest difference between the smoothed signal and the baseline normal signal (here: 0). A match is defined as a single observation for which such a deviation from zero (0) was found in both VEGA and ZEN.

*Precision* (positive predictive value) is defined as
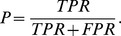

*Recall* (sensitivity) is defined as



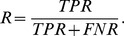

*F-scores* (harmonic mean, interpreted as a weighted average of precision and recall) are given by the combination of P and R:



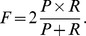
We present results for method comparison on the simulation data, cross-validation effectiveness and convergence. They are summarized in Table 1. Note that for the F-scores, 1 =  best performance and 0 =  worst performance. The best performing method is indicated in bold font. It can be seen that for no and very little amount of noise (0.1), performance for the 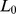 norm and VEGA are equivalent. Increasing the noise levels VEGA seems to perform slightly better. For noise level 0.6, VEGA wins for precision, but not for Recall and F-score. For even higher levels of noise, there is no clear winner. However, these levels of noise are not very interesting, since real-life data of this quality would not be analyzed.

### Applications

In this section we discuss two applications: smoothing of CNV signals (as in the above study) and scatterplot smoothing combined with segmented mixture estimation. The data were obtained in the Erasmus University Medical Center and concern several types of brain tumors [20]. In the examples below, we use tumor samples named GBM 139.CEL, GBM 180.CEL, GBM 203-2.CEL and GBM 254.CEL. Since this research focuses largely on chromosome 9, we only use signals on this chromosome in our illustrations.

Figure 7 shows smoothing of copy number variations in GBM 139.CEL, using odd-even cross-validation to select a good 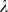. There is not much to say about this result: the segmentation conforms to our visual impression of what the data tell us. Remarkable is the rather narrow segment at 28 MB that is detected.

ZEN smoothing of the allelic ratio in GBM 139.CEL is shown in Figure 8. Most segment boundaries, but not all, correspond to those found in the copy number signal.

Although ZEN performance was already addressed, we also compared our copy number results to results from dedicated copy number software, CNAG [16]. In Figure 9 we show copy number maps for selected interesting regions on chromosome 9, and we show the corresponding segmented allelic imbalance map for the four samples mentioned above. In Figure 10 it shows that CNAG provides equivalent results on the same selected regions, but with less noise in the ZEN smoother. Therefore, we argue that ZEN outperforms VEGA and CNAG.

The adaptive weights in the penalty are small where jumps occur, and so they indicate segment boundaries. This was done to produce Figure 11, where histograms and estimated normal mixtures are shown. The package mclust was used to estimate the mixtures. It chooses the number of components (which we limited to maximally four) based on BIC. Apparently the two components of the mixture in the top-right panel have longer tails than a normal distribution, and mclust uses the sum of a narrow and a wide normal distribution to approximate them.

## Discussion

Smoothing algorithms generally have two components: one to measure the fidelity to the data, the other a penalty on roughness of the result. For the first term typically a sum of squares or of absolute values of residuals (i.e. data minus fit) is being used. To measure roughness, the size of the differences between adjacent fitted values is an effective and attractive choice. The way these differences are being expressed has a large influence on the shape of the fitted curve. [1] showed that a variant of the Whittaker smoother, using the 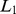 norm in the penalty on differences, is attractive for copy number smoothing, because it can deliver constant segments with relatively sharp jumps in between.

We propose to use the 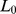 norm, essentially the count of the number of jumps. To make computation practical, we also present an algorithm based on iteratively re-computed weights in a sum-of-squares penalty. This turns out to be effective: very sharp jumps between segments are obtained.

Because our algorithm can automatically interpolate missing data, it is possible to use a simple odd-even scheme for cross-validation, to automatically choose the amount of smoothing. However, we propose cross-validation only as a guide to find a good ball park for the penalty parameter, because fast and easy visualization is our main goal.

We use cross-validation-based smoothing to compare classification performance in a little contest with VEGA, using the simulated data that come with that software. The performance of our smoother is quite close to that of VEGA. This should give users the confidence that the segments they get are realistic ones.

The objective function of the proposed smoother is non-convex. In principle this is a cause for worries: we can never be sure that the global minimum was found. In practice we have seen that we always get very good results, as judged by visual inspection. To give some insight, we presented a few illustrations of how intermediate results converge towards the final solution.

A plot of copy numbers along a chromosome contains only one “curve” as a noisy band with jumps. A plot of allelic imbalance is different: at any position from one to three bands can be present. Jumps are present too and there the number of bands as well as their positions can change. The smoothing algorithm for copy numbers will not work on such data. Instead we modified the scatterplot smoother of [2], which is based on smoothing rows and columns of a two-dimensional histogram by penalized least squares. One of the penalties was changed, to accept iteratively recomputed weights, like in the copy number smoother. The weights are based on summaries of the columns of the histogram, to have the same segment boundaries in all rows. The approach is rather ad-hoc, as there is no explicit objective function to minimize, but the results look attractive and computation is fast, allowing interactive use.

Segmented smoothing of allelic imbalance can indicate boundaries that are not visible in copy numbers. An example is copy number-neutral loss of heterozygosity. It makes sense to study histograms of the (log of the) allelic ratio for each separate segment in the plot. In addition to histograms we also propose fitting of mixtures of normal distributions. The package mclust gives good results.

In summary, we believe that we have extended the toolbox for exploration of copy number variation and allelic imbalance with attractive new instruments. All computation was done in R [21] and the programs are available from the first author on request.

## References

[1] Eilers P, DeMenezes R (2005). Quantile smoothing of array cgh data.. Bioinformatics.

[2] Eilers P, Goeman J (2004). Enhancing scatterplots with smoothed densities.. Bioinformatics.

[3] Liu Z, Li A, Schulz V, Chen M, Tuck D (2010). Mixhmm: Inferring copy number variation and allelic imbalance using snp arrays and tumor samples mixed with stromal cells.. PLoS ONE 5.

[4] Wang K, Li M, Hadley D, Liu R, Glessner J (2007). Penncnv: an integrated hidden markov model designed for high-resolution copy number variation detection in whole-genome snp genotyping data.. Genome Res.

[5] Colella S, Yau C, Taylor J, Mirza G, Butler H (2007). Quantisnp: an objective bayes hiddenmarkov model to detect and accurately map copy number variation using snp genotyping data.. Nucleic Acids Res.

[6] Morganella S, Cerulo L, Viglietto G, Ceccarelli M (2010). Vega: Variational segmentation for copy number detection.. Bioinformatics.

[7] Muggeo V, Adelfio G (2011). Efficient change point detection for genomic sequences of continuous measurements.. Bioinformatics.

[8] Budinska E, Gelnarova E, Schimek M (2009). Msmad: a computionally efficient method for the analysis of noisy array cgh data.. Bioinformatics.

[9] Tibshirani R (1996). Regression shrinkage and selection via the lasso.. J Royal Statist Soc B.

[10] Lai W, Johnson M, Kucherlapati R, Park P (2005). Comparative analysis of algorithms for identifying amplifications and deletions in array cgh data.. Bioinformatics.

[11] Marenne G, Rodriguez-Santiago B, ClosasM, Perez-Jurado L, Rothman N, et al (2011). Assessment of copy number variation using the illumina infinium 1m snp-array: a comparison of methodological approaches in the spanish bladder cancer/epicuro study.. Hum Mutat.

[12] Winchester L, C Y, J R (2009). Comparing cnv detection methods for snp arrays.. Brief Funct Genomic Proteomic.

[13] Tsuang D, Millard S, Ely B, Chi P, Wang K (2010). The effect of algorithms on copy number variant detection.. PLoS ONE.

[14] Zhang D, Qian Y, Akula N, Alliey-Rodriguez N, Tang J (2011). Accuracy of cnv detection from gwas data.. PLos ONE.

[15] Bengtsson H, Ray A, Spellman P, Speed T (2009). A single-sample method for normalizing and combining full-resolution copy numbers from multiple platforms, labs and analysis methods.. Bioinformatics.

[16] Nannya Y, Sanada M, Nakazaki K, Hosoya N, Wang L (2005). Robust algorithm for copy number detection using high-density oligonucleotide single nucleotide polymorphism genotyping arrays.. Cancer Res.

[17] Eilers P (2003). A perfect smoother.. Anal Chem.

[18] Schlossmacher E (1973). An iterative technique for absolute deviations curve fitting.. Journal of the American Statistical Association.

[19] Fraley C, Raftery A (2004). Model-based methods of classification: Using the mclust software in chemometrics.. Journal of Statistical Software 18.

[20] Bralten L, Kloosterhof N, Gravendeel L, Sacchetti A, Duij E (2010). Integrated genomic profiling identifies candidate genes implicated in glioma-genesis and a novel leo1-slc12a1 fusion gene.. Genes, Chromosomes and Cancer.

[21] R Development Core Team (2011). R: A language and environment for statistical computing.. R Foundation for Statistical Computing.

